# Efficacy Assessment of Biological Treatments in Severe Asthma

**DOI:** 10.3390/jcm14020321

**Published:** 2025-01-07

**Authors:** Daniel Laorden, Javier Domínguez-Ortega, David Romero, Elena Villamañán, Pablo Mariscal-Aguilar, Paula Granda, Santiago Quirce, Rodolfo Álvarez-Sala, on behalf of ASMAGRAVE-HULP Group

**Affiliations:** 1 Department of Pneumology, Hospital La Paz, Universidad Autónoma de Madrid, IdiPAZ, and CIBER of Respiratory Diseases, 28046 Madrid, Spain; 2 Department of Allergy, Hospital La Paz, IdiPAZ, and CIBER of Respiratory Diseases, 28046 Madrid, Spain; 3 Department of Pharmacy, Hospital La Paz, IdiPAZ, 28029 Madrid, Spain; 4 Pharmacy Department, Gómez Ulla Military Hospital, 28047 Madrid, Spain

**Keywords:** severe asthma, biologic, efficacy tools, biomarkers, personalized medicine

## Abstract

Uncontrolled, severe asthma remains a significant clinical challenge, affecting a small proportion of asthma patients worldwide. Despite advancements in treatment options, a subset of patients continues to experience frequent exacerbations, uncontrolled symptoms, and impaired quality of life. The advent of biological therapies has revolutionized the management of severe asthma, offering targeted treatments that address specific inflammatory pathways. This review provides a comprehensive overview of the efficacy and response criteria of biological treatments in severe asthma, focusing on clinical, functional, and inflammatory markers used to help in the evaluation of the biologic treatment. Key response criteria include symptom control, reduction in exacerbations, improvement in lung function, and a reduction in or the discontinuation of oral corticosteroids. Biomarkers such as blood eosinophils and exhaled nitric oxide (FeNO) are essential tools in guiding treatment adjustments. Real-world studies underscore the importance of personalized treatment strategies, as variability in response to biological therapies can be significant. The emergence of tools such as the FEOS score and EXACTO questionnaire offer quantitative measures for assessing biological response and guiding clinical decisions. Additionally, predictive factors for better or poorer responses, such as pre-treatment lung function and comorbidities, like obesity and rhinosinusitis, are critical in patient selection. This review highlights the need for ongoing reassessments and potential modifications of therapy in cases of suboptimal response. Practical considerations for switching biological therapies are discussed, emphasizing the importance of tailoring treatments to individual patient profiles and disease phenotypes. With the continued development of personalized medicine, the outlook for patients with severe asthma is improving, selecting specific biomarkers to improve the selection of the biologic treatment.

## 1. Introduction

Severe uncontrolled asthma [1,2] remains a challenging condition to manage, affecting a small but significant proportion of asthma patients worldwide, approximately 5% in Europe [1]. Despite advancements in therapy and the understanding of asthma pathophysiology, a subset of patients continues to experience uncontrolled symptoms, frequent exacerbations, and a diminished quality of life. The advent of biological therapies has marked a pivotal shift in the management of severe asthma, offering targeted treatments that address specific inflammatory pathways involved in the disease process [1,2].

The management of severe asthma is driven by asthma guidelines [1,2] and consensus documents on asthma [3], which provide a framework for the diagnosis, treatment, and monitoring of asthma, emphasizing the importance of a personalized approach to therapy. Severe asthma is defined as asthma that remains uncontrolled despite adherence to high-dose standard therapy and the consideration of comorbid conditions that can mimic asthma symptoms.

Assessing the response to these treatments is crucial for optimizing patient outcomes and involves a comprehensive evaluation of clinical, functional, and inflammatory markers. In patients with type 2 severe asthma, characterized by eosinophilic or phenoendotypes, monoclonal antibodies have been developed to block key cytokines such as IL-5, IL-4, IL-13, and immunoglobulin E (IgE) [4]. For instance, mepolizumab and reslizumab target IL-5, reducing eosinophil proliferation and activation, while benralizumab acts on the IL-5 receptor, inducing eosinophil apoptosis. Dupilumab inhibits the shared receptor for IL-4 and IL-13, thereby attenuating airway inflammation and improving lung function. Omalizumab, however, targets IgE, preventing its binding to high-affinity receptors on mast cells and basophils, thereby limiting allergic responses. More recent therapies, such as tezepelumab, act upstream on epithelial-derived alarmins like TSLP, offering benefits even in patients with low type 2 inflammation. These therapies enable a more personalized approach to severe asthma management, guided by the patient’s phenotype and biomarkers [4]. However, other therapies such as risankizumab and fevipiprant (anti-interleukin-23 monoclonal antibody and oral prostaglandin D2 receptor 2, respectively) did not provide clinical benefits in patients with severe asthma [4]. Itepekimab, a monoclonal antibody that inhibits interleukin-33 (IL-33), was demonstrated to prevent asthma control loss and improve lung function in phase 2 clinical trials with moderate-to-severe asthma patients [4,5]; nevertheless, further research in phase 3 trials is necessary to confirm the efficacy and safety of itepekimab in a larger population of asthma patients [5].

This review provides an overview of the literature on biological therapy response evaluation and offers tools and strategies to objectively modify these treatments. The methodology employed in this review was a literature search conducted on the PubMed database, spanning from 1 January 2017 to 20 December 2024. The keywords used for the search were “severe asthma”; “biologic”; “Efficacy tools”; “Biomarkers”, and “Personalized medicine”. We included 43 papers published in English that discussed the evaluation of biologic therapy. The inclusion criteria were papers that discussed the different tools and techniques that measure the biological response, such as the FEOS score [6], EXACTO [3], and Upham super-responder criteria [7]. Studies must contain clear information about the response to biologic treatment in asthma and remission. We excluded papers not directly related to the issue of this review and studies not published in English, abstracts, and commentary articles.

## 2. Response Criteria to Asthma Treatment

The consensus documents, including updates in 2020 and 2022 [3,8] and the latest guidelines [1,2], recommend the regular monitoring of the disease’s evolution and therapeutic response, highlighting the variable nature of asthma and the need for individualized treatment plans. They propose a multifaceted assessment of response to therapy, encompassing clinical response, functional improvement, reduction in exacerbations, and inflammatory marker levels after four months, and the evaluation of exacerbations at twelve months regarding seasonal crises. The consensus emphasizes the role of biomarkers such as eosinophil counts and exhaled nitric oxide (FeNO) measurements in guiding treatment adjustments and the potential indication for biological therapies in patients with eosinophilic or allergic asthma refractory to inhaled treatments.

The evaluation of treatment response in severe asthma encompasses several domains:

- Symptom control: The utilization of questionnaires, such as the Asthma Control Test (ACT), the Asthma Control Questionnaire (ACQ), and Asthma Impairment and Risk Questionnaire (AIR-Q), provides subjective measures of symptom control. An ACT score of less than 20, an ACQ score greater than 1.5, and an AIRQ score higher than 7 indicate poor control. It is also important to assess the quality of life in patients with asthma. The Asthma Quality of Life Questionnaire (AQLQ) provides clinicians with essential information regarding symptoms, daily activities, emotional well-being, and environmental exposure. A score below 5 signifies a poor quality of life.
- Reduction in severe exacerbations: A significant reduction in the number of severe asthma exacerbations, especially those that require systemic corticosteroids, emergency department visits, or hospitalizations, is a key indicator of treatment success.
- Maintenance of corticosteroid use: An ideal response would involve a reduction in or discontinuation of oral corticosteroid use, considering the adverse effects associated with long-term corticosteroid therapy.
- Lung function tests: Improvements in forced expiratory volume in 1 s (FEV_1_) and the FEV_1_/FVC ratio are objective measures of pulmonary function improvement.
- Inflammatory markers: Eosinophil counts in blood and sputum, along with exhaled nitric oxide (FeNO) levels, serve as biomarkers of inflammation and treatment response.
- Adherence and comorbidities: Ensuring patient adherence to both biological therapy and inhaled corticosteroids is crucial. E-prescriptions can be an important support for evaluating adherence. Additionally, the management of comorbidities plays a significant role in the overall response to therapy. A recent study showed different expressions of microRNAs between asthmatic obese patients and healthy ones, suggesting microRNAs’ specific involvement in the regulation of the lungs’ inflammatory response with promising potentials for asthma clinical evaluations and management [9].

The consensus documents and guidelines propose a period of four to twelve months to evaluate the response to biological therapies adequately [1,3,8]. This timeframe allows for an assessment of clinical improvement, reduction in exacerbation frequency, and changes in lung function and inflammatory markers. The Spanish Asthma Guideline [2] recommends a period of four to six months to evaluate biologic response, assessing asthma symptoms, questionnaires, the number and severity of exacerbations, and pulmonary functional test and FEOS scores [6] as principal points. If there is an adequate response, it recommends continuing the biological treatment, but if there is an inadequate response, it suggests re-evaluating the phenotype, identifying the possible causes of biological failure, and switching to another biologic if necessary.

The first Severe Asthma Spanish Consensus [8] stated the parameters that were important to focus on to evaluate the biological response (symptoms, exacerbations, oral corticosteroids, and FEV_1_), and it classified these parameters depending on complete response, control, partial response, and non-response. The second severe asthma consensus [3] included the EXACTO score. This questionnaire assigns a score to any of the parameters that were presented in the previous consensus (symptoms, exacerbations, oral corticosteroids, and FEV_1_) (Table 1). The final score classifies the biologic response as complete response/super-responder, good response, partial response, or non-response (Table 1).

Upham et al. [7] published super-responder criteria for biologic treatment, based on a Delphi methodology with asthma experts in Pulmonology and Allergology. They defined what is considered a very good responder to biologic treatment. They described six criteria based on the following parameters: symptoms, exacerbations, oral corticosteroids, and FEV_1_ (Table 2). Three of these were major criteria and three minor criteria. The authors state that to be considered a super-responder, a patient must have three criteria, including two major criteria (Table 2).

Another tool was created to measure the biologic response to biologic drugs named the ’FEOS score’ [6]. Its name comes from the four parameters that it is based on: symptoms, exacerbations, oral corticosteroids, and FEV_1_ (Table 3). The FEOS score measures improvement or worsening in any of the four parameters mentioned previously and assigns a score to each parameter depending on the improvement or worsening after the prescription of biologics. The score assigned to every parameter is based on a Delphi methodology with asthma experts in Pulmonology and Allergology. They assigned higher scores to the variables that were considered more important to evaluate biologic response (Table 3). There is also an online tool used to calculate the FEOS score easily [10]; it helps the clinician to sum the four parameters faster and make a faster and objective decision to continue or remove the biologic drug.

## 3. Real-Life Measurement of Biologic Response

### 3.1. Super-Responder, Responder, and Non-Responder Criteria

Several studies have been undertaken to measure the response to biological therapies (Table 4) [11]. It would be advisable to unify all criteria to clearly define what is considered a super-responder, good responder, non-responder, or remission [11].

The definition of a clinically significant response varies but generally includes improvements in symptom control (ACT score higher than 20), reduction in exacerbations (one or fewer total exacerbations), and a reduction in or withdrawal of oral corticosteroids (less than 5 milligrams of prednisone). Most patients in real-world studies met at least one of these criteria (Table 4), demonstrating the efficacy of biological therapies across a range of severity and phenotypes in severe asthma. These criteria help clinicians to objectively evaluate the response and assist in the decision to maintain or modify the biological treatment.

There are several criteria for measure the response; however, Upham criteria [7] are the most accepted for measuring the optimal response (super-response) to biologic treatment. In Table 4, we can observe the results of the response to biological treatment. Valverde-Monge et al. [11] achieved 91% of patients meeting response criteria, and 55% of patients met the Upham super-responder criteria [7] with a mean follow-up of 55 ± 38.8 months. Estravís et al. [12] obtained super-response in 59% of patients after one year. Pérez de Llano et al. [13] analyzed the number of super-responders in patients treated with reslizumab, observing a rate of 54% of super-responders after one year. Laorden et al. [14] observed 50% super-responders and 45% responders to anti-IL5 after one year. These good results improved after two years (63% super-responders and 28% responders) and after three years (72% super-responders and 22% responders).

There are several real-life studies evaluating omalizumab response. The most important study is a meta-analysis [18] of 86 publications with a duration of omalizumab treatment of, at least, 16 weeks, between January 2005 and October 2018. The study demonstrated an 82% good/excellent response rate at 12 months. It demonstrated a significant improvement in FEV_1_ (250 milliliters), ACQ score (−1.13), exacerbation rates (relative ratio: 0.41), and corticosteroid use (relative risk: 0.59) at 12 months.

Mepolizumab has also demonstrated good response in previous studies: A prospective, two-year observation of mepolizumab response [15] showed a super-response in 22% of patients after one year and 24% after two years. Super-response was defined as an ACT score improvement of ≥6 points, the elimination of exacerbation, an improvement of ≥400 mL in FEV_1_, and the complete tapering of OCSs. The rate of responders was 43% after one year and 54% after two years. Response was defined as an ACT score improvement of ≥3 points, an increase of ≥200 mL in FEV_1_, and a reduction in exacerbation of ≥50% or a reduction in OCSs of ≥50%.

A study was conducted involving 72 patients over a median duration of 13 months to evaluate the effects of dupilumab [17]. A clinically significant response was defined as achieving any of the following: an improvement in the ACT score to 20 or greater, a reduction in FeNO to less than 25 ppb, or an improvement in FEV_1_ by 120 mL or more. All patients met at least one of these criteria. The key findings include an average increase in the Asthma Control Test (ACT) score by 6 points and an improvement in FEV_1_ by 181 mL. Additionally, there was a decrease in FeNO by 24 parts per billion (ppb). Notably, 20 patients who previously failed other medications responded to dupilumab. Among the nine patients on oral corticosteroids (OCSs), six were able to discontinue OCSs, and two reduced their dosage.

A retrospective study of 130 patients treated with benralizumab revealed that 39% of patients were super-responders and 86% were responders after one year [16]. Response was defined as a reduction of 50% in the annualized exacerbation rate (AER) or in OCS dose after 48 weeks of treatment. Super-response was defined as zero exacerbations and no OCSs for asthma.

These studies highlight the importance of selecting the appropriate biological therapy based on individual patient characteristics, including asthma phenotype, eosinophil counts, and prior treatment responses. However, all these studies employ different response criteria (Table 4), and Valverde-Monge et al. [11] pointed out the importance of unifying response criteria for future investigations. However, most of them only included patients who ended treatment at the time of inclusion, forgetting to collect the patients that were withdrawn the biologic treatment and leading to a possible selection bias. For this reason, prospective studies are needed to evaluate the biologic response.

### 3.2. FEOS and EXACTO Scores

There are also other tools for measuring the response to biologic treatment, such as the FEOS score or the EXACTO scale. The FEOS score has been used in several real-life studies to measure the biological response. Pérez de Llano et al. [13] obtained a mean score of 76 after one year of reslizumab treatment in a multicenter study of 193 patients. Estravís et al. [12] observed a mean score of 96 after six months of treatment in a study involving 32 patients treated with mepolizumab, benralizumab, and dupilumab. Laorden et al. [14] studied the FEOS score’s evolution during three years in 60 patients treated with benralizumab, mepolizumab, and reslizumab, achieving a median score of 76 points in the first year, 76 the second year, and 94 the third year of study. Loli-Ausejo et al. [19] obtained a median of 70 points in the FEOS score after three to five years of mepolizumab treatment in 44 patients. All these results are along the same line and indicate that the FEOS score could be an interesting tool to use to quantitatively measure the biologic response. There is also another study led by Chiner et al. [20] involving 58 patients treated with benralizumab during 12 months with a FEOS score mean of 73 ± 14.

Laorden et al. [14] aimed to find a cutoff point in the FEOS score [6] to define super-responders using the Upham criteria [7]. After a Receiver Operating Characteristic (ROC) Curve calculation, a FEOS score of 75 or higher could be used as a cutoff point to define super-responders. The authors point out that the FEOS score [6], with its quantitative measure of the response, could be more precise in measuring the response than qualitative responder criteria, which only indicate whether the patient is a responder or not. Thus, the FEOS score [6] could be obtained easily by a new online calculator [10] that can be used in clinical consultation, providing the FEOS score automatically with no need to sum all the scores. The FEOS score could be a practical tool that helps us make more objective decisions in our daily clinical practice.

Estravís et al. [21] point out the great importance of normalizing the FEOS score [6] because patients have different baseline characteristics. For example, a patient who does not use OCSs before biologics will always have a lower potential absolute FEOS score than others with OCS use. This is why they propose normalizing the FEOS score to correctly evaluate the biological response.

The EXACTO questionnaire [3] is a quantitative tool that becomes qualitative by classifying patients as complete/super-responders, good responders, partial responders, or non-responders depending on the final score. There are few studies published on the EXACTO [3] evaluation of response; however, Chiner et al. [20] studied it in 58 patients treated with benralizumab after 12 months, obtaining 57% complete response, 28% good response, and 15% partial response.

### 3.3. Predictive Factors of Response

In evaluating the potential factors of a super-response or higher score in FEOS [6], Pérez de Llano et al. [22], in their study of 414 patients treated with omalizumab, mepolizumab, reslizumab, benralizumab, and dupilumab, pointed out that higher FEV_1_, or a longer duration of treatment could be positive factors for better response. These conclusions are along the same line as the previous studies mentioned, Valverde-Monge et al. [11] and Laorden et al. [14]. Valverde-Monge et al. [11] also found that higher ACT test results and the presence of nonsteroidal anti-inflammatory drugs (NSAIDs) exacerbated respiratory disease as better biologic predictors, and Laorden et al. [14] observed older age as a potential beneficial factor for a better response; these results are in line with the previously mentioned papers [11,13,23].

There are also other studies that evaluate the risk factors for a poorer response. Valverde-Monge et al. [11] found that obesity is a risk factor for suboptimal response. Pérez de Llano et al. [22] agreed with these findings and pointed out that male sex is another factor for poorer response. Valverde-Monge et al. [11] added that the presence of previous intensive care unit (UCI) admissions and a higher number of exacerbations are predictors of poorer response.

These factors associated with a better or poorer response could be markers for prescribing the biological treatment. However, we still need bigger populational studies or meta-analyses that could determine which type of biological treatment is associated with the suboptimal or super-response.

### 3.4. Remission

The remission concept in asthma has been reintroduced again once the new biologic therapies demonstrate the potential modification of the disease. Menzies-Gow et al. [24] propose four remission criteria for clinical remission, including asthma symptoms, no use of OCSs, the stabilization and optimization of lung function, and patient agreement. For complete remission, they add two criteria: improvement in asthma biomarkers and negative hyper-responsiveness. This remission can be achieved on-treatment or off-treatment. Rial and Dominguez-Ortega [25] introduced the concept of inflammatory remission that includes negative airway or serum biomarkers, permitting variability in obstruction and airway hyper-responsiveness.

In 2023, Oishi et al. [23] performed a study evaluating clinical, inflammatory deep/complete remission in 54 patients, obtaining clinical and immunological remission in 68% of patients and 31% of patients that achieved deep/complete remission after one year of treatment. Correa-Borit et al. [26] achieved 58% and 63% remission after three and four years of mepolizumab treatment. Predictive factors for deep remission were a shorter asthma duration, higher previous FEV_1_, and adult-onset asthma; these results are in line with other predictive good responses mentioned previously in this manuscript and could be due to minor asthma remodeling in the airways. The latest Spanish Consensus on Remission in Asthma (REMAS) [27] adds the item of no bronchial remodeling lesions on imaging tests to achieve complete remission and states that these characteristics must be present at least for three years.

## 4. Approaches to Managing Suboptimal Response

The exploration of real-world efficacy and comprehensive criteria for assessing treatment response in severe asthma underlines the transformative impact of biological therapies. However, the management of patients who exhibit a suboptimal response poses a significant challenge, necessitating a strategic approach to reassessment and potential therapy adjustment. This document will continue to delve into strategies for managing suboptimal responses to biological therapy based on clinical guidelines [2,28] and position papers [3,29], emphasizing the importance of a meticulous reassessment process, potential modifications to treatment, and considerations for changing biological agents.

In instances where patients exhibit a suboptimal response to biological therapy, a thorough calculation of the patient’s condition, treatment adherence, and potential external factors is essential. This reassessment process aims to identify the underlying reasons for the inadequate response and to guide subsequent treatment adjustments.

### Confirmation and Management Strategies for Suboptimal Response

The initial steps should involve verifying the patient’s adherence to the prescribed biological and inhaler therapy and correct self-administration techniques [2]. Exposure to allergens, smoking, and other environmental or lifestyle factors can influence asthma control [2].

Given that up to 90% of patients with severe asthma have at least one comorbidity, assessing the control and impact of these existing or new comorbid conditions is vital. Conditions such as obesity and obstructive sleep apnea, gastro-esophageal reflux disease (GERD), rhinosinusitis with nasal polyposis, anxiety, or vocal cord dysfunction can exacerbate asthma symptoms and interfere with treatment efficacy. There is an investigation [30] that concludes that 70% of partial response to biologic therapy is due to comorbidities. If rhinosinusitis with nasal polyposis is detected, there are some biologic treatments that have demonstrated efficacy in controlling this disease, such as mepolizumab [31,32] and dupilumab [33,34]. There are some studies [29,35] that suggest that patients with COPD experience worse outcomes compared to those with asthma, with a higher frequency of exacerbations.

Conditions such as eosinophilic granulomatosis with polyangiitis, allergic bronchopulmonary aspergillosis, chronic eosinophilic pneumonia, hypereosinophilic syndrome, and eosinophilic bronchiolitis should be considered. Comprehensive diagnostic measures including CT scans, blood tests, and complete functional tests are necessary to exclude these disorders [2,29].

Additionally, it is important to rule out chronic bronchial infections caused by bacteria or fungi through sputum cultures and bronchoscopy. Cosío et al. [36] have demonstrated the potential of bronchoscopy in evaluating suboptimal responses, with a high prevalence of laryngeal, tracheal, mucosal pathology and bronchial infection in 100 patients studied with bronchoscopy which may develop into a suboptimal biologic response.

Differentiating between inflammatory exacerbations and infectious exacerbations is very important [29,37]. Inflammatory exacerbations may indicate the need for biological change, and infectious exacerbations may require treatments such as azithromycin.

Re-evaluating phenotype dominance is very important; asthma is a heterogeneous disease, and patients may experience changes in their asthma phenotype over time because of different exposure or patient causes, including the current biological therapy [2,29]. Eosinophil levels, allergies, and other asthma markers must be obtained to assess the cause of the suboptimal response. It is important to consider the dichotomy between blood eosinophils and lung eosinophils (evaluated through induced sputum or bronchoscopy) when evaluating the phenotype [2,29]. Additionally, measuring interleukin-5 or other inflammatory markers in exhaled air could be a promising new advancement for evaluating the asthma phenotype [38,39]. We have to consider that biomarkers can be influenced by treatments (such as corticosteroids or biological therapies) or environmental factors (like tobacco smoke or allergens), which may lead to the incorrect selection of biological treatments.

One of the challenges observed in clinical practice is insufficient dosing, particularly in patients with elevated body mass index (BMI), which would require adjustments based on weight. Specifically, reslizumab is a drug that requires the adjustment of doses by weight [2,29,40,41]. Also, the intravenous administration of the drug allows for a faster onset of action, achieving 100% bioavailability. Monitoring drug levels could become essential in ensuring therapeutic efficacy [29].

In some cases, the development of anti-drug antibodies has been observed; however, the study of this phenomenon is not well standardized, as has been studied in patients with rheumatological diseases. Clinically, this can manifest as a late poor response after an initial good response, suggesting the need for further investigations into the underlying mechanisms and appropriate therapeutic adjustments [2,29].

Autoimmune phenomena can be complex and multifactorial. In some cases, eosinophilic granulomatosis with polyangiitis (EGPA) may be masked by prior treatment, such as corticosteroids or anti-IL5 treatment [29]. We have to be careful with potential hypereosinophilia after anti-IL4/IL13 blockades, where eosinophil migration is reduced, leading to accumulation in the bloodstream. This process inhibits eotaxin-3, VCAM-1, and TARC without suppressing eosinopoiesis, resulting in transient eosinophilia [42]; there is one clinical case of autoimmune phenomena that developed after anti-IL4/IL13 blockades [43].

Adverse events associated with treatment (myalgia, fatigue, or arthralgia) can sometimes be misinterpreted as poor therapeutic response. Corticosteroid tapering should be closely monitored for signs of adrenal insufficiency, including cortisol levels, and the emergence of conditions previously controlled by corticosteroids, such as allergic bronchopulmonary aspergillosis (ABPA) and eosinophilic granulomatosis with polyangiitis (EGPA) [2,29].

Mucus plugs are another critical factor, affecting approximately 58% of asthma patients. Anti-T2 therapies, including anti-IL13, anti-IL5/R, and anti-TSLP, have been shown to improve ventilation and reduce mucus plugs. This reduction correlates with improvements in pulmonary function, leading to a reduction in dyspnea, cough, and chest tightness in affected patients [29].

Implementing more frequent monitoring and providing additional patient education and support can help improve adherence, asthma control, and comorbidities. This might include the use of digital health tools, asthma education programs, and regular follow-up appointments to reassess symptoms and adjust treatment as needed [2].

## 5. Practical Considerations to Change the Biological Therapy

Changing biological therapies is a decision that should not be taken without objective parameters and requires a careful consideration of the available evidence and the patient’s clinical history and preferences. The counsel of an expert multidisciplinary asthma unit formed by an allergist, pulmonologist, pharmacist, otorhinolaryngologist, psychologist, and other specialists is the cornerstone for finding the best option for the patient’s condition. Studies and clinical experiences have shown that switching biological therapies can lead to improvements in asthma control, symptom reduction, and decreased exacerbation rates for certain patients. For instance, patients transitioning from omalizumab to an anti-IL-5/R monoclonal antibody have demonstrated significant improvements in clinical outcomes in selected cases where the initial treatment failed to achieve the desired control [44,45].

The choice of the next biological therapy should be informed by an understanding of the underlying mechanisms driving the patient’s asthma and the specific targets of available biological treatments. For example, for a patient with eosinophilic asthma not responding adequately to an anti-IL-5 therapy, considering an anti-IL-4/IL-13 therapy such as dupilumab might be rational due to its broader action on the type 2 inflammation pathway [11,46].

It is necessary to consider the patient’s preferences, potential for adherence, and any previous experiences with biological therapies. The safety profile and tolerability of the new biological therapy should also be evaluated, considering any comorbid conditions that might influence the risk–benefit analysis. The availability and cost of biological therapies can vary significantly, which may influence the decision-making process. Clear criteria should be established for assessing the response to the new therapy, including symptom control, exacerbation rates, lung function tests, and biomarker levels.

**Figure 1:**
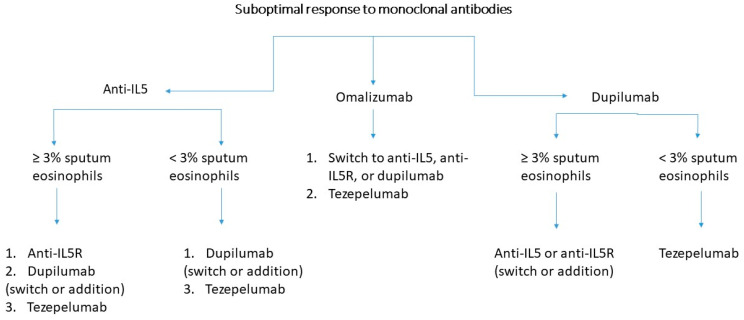
Algorithm for switching biologics. Extracted and modified from Pérez de Llano et al. [29].

We recommend following the algorithm for switching biologics (Figure 1) proposed by Pérez de Llano et al. [29] to guide clinical practice.

## 6. Conclusions

The management of severe asthma, particularly regarding the use of biological therapies, is a complex and dynamic process that requires the ongoing assessment and individualization of treatment strategies. For patients with a suboptimal response to initial biological therapy, a systematic approach to reassessment and management can help identify the most appropriate course of action, whether it involves addressing modifiable factors, optimizing the current therapy, or switching to a different biological agent. The advancement of personalized medicine and the availability of multiple targeted therapies are improving disease control and the quality of life of patients.

## Figures and Tables

**Table 1:** Parameters for evaluating biological response in the last severe asthma consensus in 2022, the EXACTO scale. Extracted and adapted from Alvarez-Gutiérrez FJ et al. [3].

	Exacerbations	ACT	Oral Corticosteroids	FEV_1_
**Non-response**	Same or higher = 0 points	Same or worse than initial = 0 points	Same or higher dose = 0 points	Same or worse than initial = 0 points
**Partial response**	≥2 exacerbations = 1 point	<20 and improvement < 3 = 0 points	Reduction of < 50% dose but not withdrawn = 1 point	Increase < 10% and 100 mL but < 80% = 0 point
**Good response**	≤1 exacerbation = 2 points	>20 and improvement ≥ 3 = 1 point	Reduction of ≥ 50% dose but not withdrawn = 2 points	Increase ≥ 10% and 100 mL but FEV_1_ < 80% = 1 point
**Complete response**	No exacerbation = 3 points	≥20 points = 2 points	Withdrawn or doses ≤ 5 mg/day of prednisone = 3 points	Increase ≥ 10% and 100 mL but FEV_1_ ≥ 80% = 2 points
		**Score Without OCSs**		**Score with OCSs**
**Non-response**		0–1		0–2
**Partial response**		2–4		3–6
**Good response**		5–6		7–9
**Complete response**		7		10

ACT: Asthma Control Test; FEV_1_: forced expiratory volume in 1 s.; OCSs: oral corticosteroids.

**Table 2:** Upham et al. super-responder criteria. Extracted and adapted from Upham et al. [7].

Major Criteria	Minor Criteria
Exacerbation elimination	75% exacerbation reduction
Major improvement in asthma control (≥2x the minimal clinically important difference)	Well-controlled asthma (Asthma Control Questionnaire score < 1 or Asthma Control Test score > 19)
Cessation of maintenance oral corticosteroids (or weaning to adrenal insufficiency)	≥500 mL improvement in FEV_1_
Improvement should involve three or more criteria (at least two of which should be major criteria) and should be assessed over 12 months.	

FEV_1_: forced expiratory volume in 1 s.

**Table 3:** FEOS score. Extracted and adapted from Pérez de Llano et al. [6].

Criteria	Points
**Maintenance systemic corticosteroid dose: change with respect to baseline**	
Increase	0
No change	14
Reduction < 50%	24
Reduction of 50% to 100%	29
Complete withdrawal	38
**Severe exacerbations: change with respect to baseline**	
Increase	0
No change	11
Reduction <50%	22
Reduction of 50% to 100%	27
100% reduction	38
**ACT: change with respect to baseline**	
ACT total score decrease	0
<3 point increase	5
≥3 point increase, but total score < 20 points	9
ACT ≥ 20 points	13
**Prebronchodilator FEV_1_: change with respect to baseline**	
>100 mL decrease	0
No change or change <100 mL and 10% increase	5
≥100 mL increase and 10%, but <80%	9
FEV_1_ ≥ 80%	11

ACT: Asthma Control Test; FEV_1_: forced expiratory volume in 1 s.

**Table 4:** Real-world studies measuring the biological response.

	Responders	Super-Responders	Non-Responders	Remission
Valverde-Monge et al. [11]	91.1%	55%	8.9%	27%
Estravís et al. [12]	Not analyzed	59%	41%	Not analyzed
Pérez de Llano et al. [13]	Not analyzed	54%	46%	Not analyzed
Laorden et al. [14]	45%	50%	5%	Not analyzed
Kallieri et al. [15]	43%	22%	35%	Not analyzed
Kavanagh et al. [16]	86%	39%	14%	Not analyzed
Nowsheen S et al. [17]	100%	Not analyzed	0%	Not analyzed

## References

[1] (2024). Global Initiative for Asthma; Diagnosis and Management of Difficult-to-Treat and Severe Asthma. www.ginasthma.org/severeasthma/.

[2] (2024). Spanish Asthma Guideline. Guía Española para el Manejo del Asma. GEMA 5.4. www.gemasma.com.

[3] Alvarez-Gutiérrez F.J., Blanco-Aparicio M., Casas-Maldonado F., Plaza V., González-Barcala F.J., Carretero-Gracia J.Á., Castilla-Martínez M., Cisneros C., Diaz-Pérez D., Domingo-Ribas C. (2022). Documento de consenso de asma grave en adultos. Actualización 2022. Open Respir. Arch..

[4] Brusselle G.G., Koppelman G.H. (2022). Biologic Therapies for Severe Asthma. N. Engl. J. Med..

[5] Wechsler M.E., Ruddy M.K., Pavord I.D., Israel E., Rabe K.F., Ford L.B., Maspero J.F., Abdulai R.M., Hu C.-C., Martincova R. (2021). Efficacy and Safety of Itepekimab in Patients with Moderate-to-Severe Asthma. N. Engl. J. Med..

[6] Pérez de Llano L., Dávila I., Martínez-Moragón E., Domínguez-Ortega J., Almonacid C., Colás C., García-Rivero J.L., Carmona L., García de Yébenes M.J., Cosío B.G. (2021). Development of a Tool to Measure the Clinical Response to Biologic Therapy in Uncontrolled Severe Asthma: The FEV_1_, Exacerbations, Oral Corticosteroids, Symptoms Score. J. Allergy Clin. Immunol. Pract..

[7] Upham J.W., Le Lievre C., Jackson D.J., Masoli M., Wechsler M.E., Price D.B., Mansur A., Detoraki A., Altraja A., James A. (2021). Defining a Severe Asthma Super-Responder: Findings from a Delphi Process. J. Allergy Clin. Immunol. Pract..

[8] Álvarez-Gutiérrez F.J., Blanco-Aparicio M., Plaza V., Cisneros C., García-Rivero J.L., Padilla A., Pérez-de Llano L., Perpiñá M., Soto-Campos G. (2020). Documento de consenso de asma grave en adultos. Actualización 2020. Open Respir. Arch..

[9] Mirra D., Cione E., Spaziano G., Esposito R., Sorgenti M., Granato E., Cerqua I., Muraca L., Iovino P., Gallelli L. (2022). Circulating MicroRNAs Expression Profile in Lung Inflammation: A Preliminary Study. J. Clin. Med..

[10] FEOS Score. https://feosscore.com.

[11] Valverde-Monge M., Sánchez-Carrasco P., Betancor D., Barroso B., Rodrigo-Muñoz J.M., Mahillo-Fernández I., Arismendi E., Bobolea I., Cárdaba B., Cruz M.J. (2024). Comparison of Long-term Response and Remission to Omalizumab and Anti-IL-5/IL-5R Using Different Criteria in a Real-life Cohort of Severe Asthma Patients. Arch. Bronconeumol..

[12] Estravís M., Pérez-Pazos J., Martin M.J., Ramos-González J., Gil-Melcón M., Martín-García C., García-Sánchez A., Sanz C., Dávila I. (2023). Quantitative and qualitative methods of evaluating response to biologics in severe asthma patients: Results from a real-world study. J. Allergy Clin. Immunol. Pract..

[13] Pérez de Llano L.A., Cosío B.G., Lobato Astiárraga I., Soto Campos G., Tejedor Alonso M.Á., Marina Malanda N., Padilla Galo A., Urrutia Landa I., Michel de la Rosa F.J., García-Moguel I. (2022). Asthma Control in Patients with Severe Eosinophilic Asthma Treated with Reslizumab: Spanish Real-Life Data. J. Asthma Allergy.

[14] Laorden D., Zamarrón E., Romero D., Domínguez-Ortega J., Villamañán E., Losantos I., Gayá F., Quirce S., Álvarez-Sala R. (2023). Evaluation of FEOS score and super-responder criteria in a real-life cohort treated with anti-IL5/IL5R. Respir. Med..

[15] Kallieri M., Zervas E., Fouka E., Porpodis K., Mitrova M.H., Tzortzaki E., Makris M., Ntakoula M., Papaioannou A.I., Lyberopoulos P. (2022). RELIght: A two-year REal-LIfe study of mepolizumab in patients with severe eosinophilic asTHma in Greece: Evaluating the multiple components of response. Allergy.

[16] Kavanagh J.E., Hearn A.P., Dhariwal J., d’Ancona G., Douiri A., Roxas C., Fernandes M., Green L., Thomson L., Nanzer A.M. (2021). Real-World Effectiveness of Benralizumab in Severe Eosinophilic Asthma. Chest.

[17] Nowsheen S., Darveaux J.I. (2021). Real-world efficacy and safety of dupilumab use in the treatment of asthma. Ann. Allergy Asthma Immunol. Off. Publ. Am. Coll. Allergy Asthma Immunol..

[18] Bousquet J., Humbert M., Gibson P.G., Kostikas K., Jaumont X., Pfister P., Nissen F. (2021). Real-World Effectiveness of Omalizumab in Severe Allergic Asthma: A Meta-Analysis of Observational Studies. J. Allergy Clin. Immunol. Pract..

[19] Loli-Ausejo D., Perdomo G., Mascaró B., Martínez-Olondris P., Sánchez-Fernández M.C., Mullol J., Valero A., Arismendi E., Bobolea I. (2023). Mepolizumab for Treatment of Severe Eosinophilic Asthma: A 5-Year Real-World Experience. J. Investig. Allergol. Clin. Immunol..

[20] Chiner E., Murcia M., Boira I., Bernabeu M.Á., Esteban V., Martínez-Moragón E. (2024). Real-Life Clinical Outcomes of Benralizumab Treatment in Patients with Uncontrolled Severe Asthma and Coexisting Chronic Rhinosinusitis with Nasal Polyposis. J. Clin. Med..

[21] Estravís M., Pérez-Pazos J., Martin M.J., Ramos-González J., Gil-Melcón M., Martín-García C., García-Sánchez A., Sanz C., Dávila I. (2023). Correspondence regarding the paper “Laorden D, Zamarrón E, Romero D, Domínguez-Ortega J, Villamañán E, Losantos I, Gayá F, Quirce S, Álvarez-Sala R. Evaluation of FEOS score and super-responder criteria in a real-life cohort treated with anti-IL5/IL5R. Respir. Med. 2023; 211: 107216.”. Respir. Med..

[22] Pérez de Llano L., Marina Malanda N., Urrutia I., Martínez-Moragón E., Gullón-Blanco J.A., Díaz-Campos R., Esquerre M.M., Mena A.H., Cosío B.G., Cisneros C. (2023). Factors associated with suboptimal response to monoclonal antibodies in severe asthma. Allergy.

[23] Oishi K., Hamada K., Murata Y., Matsuda K., Ohata S., Yamaji Y., Asami-Noyama M., Edakuni N., Kakugawa T., Hirano T. (2023). A Real-World Study of Achievement Rate and Predictive Factors of Clinical and Deep Remission to Biologics in Patients with Severe Asthma. J. Clin. Med..

[24] Menzies-Gow A., Bafadhel M., Busse W.W., Casale T.B., Kocks J.W.H., Pavord I.D., Szefler S.J., Woodruff P.G., de Giorgio-Miller A., Trudo F. (2020). An expert consensus framework for asthma remission as a treatment goal. J. Allergy Clin. Immunol..

[25] Rial M.J., Domínguez-Ortega J. (2022). Inflammatory Remission in T2 Severe Asthma. Front. Allergy.

[26] Correa-Borit J., Laorden D., Arnalich Montiel V., Quirce S., Domínguez-Ortega J. (2025). Is it possible to achieve remission in severe asthma? Retrospective analysis of a four-year response in a real-life cohort treated with Mepolizumab. J. Asthma Off. J. Assoc. Care Asthma.

[27] Álvarez-Gutiérrez F.J., Casas-Maldonado F., Soto-Campos G., Blanco-Aparicio M., Delgado J., Galo A.P., Quirce S., Plaza V., REMAS GROUP (2024). Spanish Consensus on Remission in Asthma (REMAS). Arch. Bronconeumol..

[28] Global Strategy for Asthma Management and Prevention (GINA) 2024. https://ginasthma.org/2024-report/.

[29] Pérez de Llano L., Cisneros C., Domínguez-Ortega J., Martínez-Moragón E., Olaguibel J.M., Plaza V., Quirce S., Dávila I. (2023). Response to Monoclonal Antibodies in Asthma: Definitions, Potential Reasons for Failure, and Therapeutic Options for Suboptimal Response. J. Investig. Allergol. Clin. Immunol..

[30] Eger K., Kroes J.A., Ten Brinke A., Bel E.H. (2021). Long-Term Therapy Response to Anti-IL-5 Biologics in Severe Asthma—A Real-Life Evaluation. J. Allergy Clin. Immunol. Pract..

[31] Han J.K., Bachert C., Fokkens W., Desrosiers M., Wagenmann M., Lee S.E., Smith S.G., Martin N., Mayer B., Yancey S.W. (2021). Mepolizumab for chronic rhinosinusitis with nasal polyps (SYNAPSE): A randomised, double-blind, placebo-controlled, phase 3 trial. Lancet Respir. Med..

[32] Domínguez-Sosa M.S., Cabrera-Ramírez M.S., Marrero-Ramos M.D.C., Dávila-Quintana D., Cabrera-López C., Carrillo-Díaz T., Del Rosario J.J.B. (2023). Real-Life Effectiveness of Mepolizumab in Refractory Chronic Rhinosinusitis with Nasal Polyps. Biomedicines.

[33] Fokkens W.J., Lund V.J., Hopkins C., Hellings P.W., Kern R., Reitsma S., Toppila-Salmi S., Bernal-Sprekelsen M., Mullol J., Alobid I. (2020). European Position Paper on Rhinosinusitis and Nasal Polyps 2020. Rhinology.

[34] De Corso E., Pasquini E., Trimarchi M., La Mantia I., Pagella F., Ottaviano G., Garzaro M., Pipolo C., Torretta S., Seccia V. (2023). Dupilumab in the treatment of severe uncontrolled chronic rhinosinusitis with nasal polyps (CRSwNP): A multicentric observational Phase IV real-life study (DUPIREAL). Allergy.

[35] Hekking P.-P., Amelink M., Wener R.R., Bouvy M.L., Bel E.H. (2018). Comorbidities in Difficult-to-Control Asthma. J. Allergy Clin. Immunol. Pract..

[36] Cosío B.G., Shafiek H., Mosteiro M., Iglesias A., Gómez C., Toledo-Pons N., Martinez R., Lopez M., Escribano Gimeno I., Pérez de Llano L. (2023). Redefining the Role of Bronchoscopy in the Workup of Severe Uncontrolled Asthma in the Era of Biologics: A Prospective Study. Chest.

[37] McDowell P.J., Diver S., Yang F., Borg C., Busby J., Brown V., Shrimanker R., Cox C., Brightling C.E., Chaudhuri R. (2021). The inflammatory profile of exacerbations in patients with severe refractory eosinophilic asthma receiving mepolizumab (the MEX study): A prospective observational study. Lancet Respir. Med..

[38] Thomas P.S., Lowe A.J., Samarasinghe P., Lodge C.J., Huang Y., Abramson M.J., Dharmage S.C., Jaffe A. (2013). Exhaled breath condensate in pediatric asthma: Promising new advance or pouring cold water on a lot of hot air? A systematic review. Pediatr. Pulmonol..

[39] Seifi M., Rastkari N., Hassanvand M.S., Naddafi K., Nabizadeh R., Nazmara S., Kashani H., Zare A., Pourpak Z., Hashemi S.Y. (2021). Investigating the relationship between particulate matter and inflammatory biomarkers of exhaled breath condensate and blood in healthy young adults. Sci. Rep..

[40] Cinqaero: EPAR—Product Information. https://www.ema.europa.eu/en/medicines/human/EPAR/cinqaero#:~:text=The%20active%20substance%20in%20Cinqaero,in%20the%20blood%20and%20lungs.

[41] Passarell J., Jaworowicz D., Ludwig E., Rabinovich-Guilatt L., Cox D.S., Levi M., Garin M., Fiedler-Kelly J., Bond M. (2020). Population Pharmacokinetic and Pharmacokinetic/Pharmacodynamic Modeling of Weight-Based Intravenous Reslizumab Dosing. J. Clin. Pharmacol..

[42] Olaguibel J.M., Sastre J., Rodríguez J.M., Del Pozo V. (2022). Eosinophilia Induced by Blocking the IL-4/IL-13 Pathway: Potential Mechanisms and Clinical Outcomes. J. Investig. Allergol. Clin. Immunol..

[43] Suzaki I., Tanaka A., Yanai R., Maruyama Y., Kamimura S., Hirano K., Kobayashi H. (2023). Eosinophilic granulomatosis with polyangiitis developed after dupilumab administration in patients with eosinophilic chronic rhinosinusitis and asthma: A case report. BMC Pulm. Med..

[44] Chapman K.R., Albers F.C., Chipps B., Muñoz X., Devouassoux G., Bergna M., Galkin D., Azmi J., Mouneimne D., Price R.G. (2019). The clinical benefit of mepolizumab replacing omalizumab in uncontrolled severe eosinophilic asthma. Allergy.

[45] Carstens D., Maselli D.J., Mu F., Cook E.E., Yang D., Young J.A., Betts K.A., Genofre E., Chung Y. (2023). Real-World Effectiveness Study of Benralizumab for Severe Eosinophilic Asthma: ZEPHYR 2. J. Allergy Clin. Immunol. Pract..

[46] Dupin C., Belhadi D., Guilleminault L., Gamez A.-S., Berger P., De Blay F., Bonniaud P., Leroyer C., Mahay G., Girodet P.-O. (2020). Effectiveness and safety of dupilumab for the treatment of severe asthma in a real-life French multi-centre adult cohort. Clin. Exp. Allergy J. Br. Soc. Allergy Clin. Immunol..

